# Knowledge, attitude and perception of community pharmacists towards pharmacogenomics services in northern Nigeria: a cross-sectional study

**DOI:** 10.1186/s40545-022-00435-x

**Published:** 2022-05-25

**Authors:** Usman Abubakar, Lienarrubini Subramaniam, Abdulkadir Ayinla, Mobolaji Nurudeen Ambali, Dzul Azri Mohamed Noor, Nur Aizati Athirah Daud, Hauwa Kulu Isah, Hiba A. Al-Shami

**Affiliations:** 1 Discipline of Clinical Pharmacy, School of Pharmaceutical Sciences, Universiti Sains Malaysia, 11800 USM Penang, Malaysia; 2 Department of Pharmaceutical Services, Hospital Management Board, Federal Capital Territory Administration, Garki, Abuja, Nigeria; 3 Al-Jidda Pharmacy and Store, Isa Kaita Road, Unguwar Rimi, Kaduna, Nigeria; 4 Faculty of Pharmacy, University of Derna, Derna, Libya

**Keywords:** Knowledge, Attitude, Perception, Pharmacogenomics, Community pharmacists, Nigeria

## Abstract

**Objectives:**

To evaluate knowledge, attitude and perception of community pharmacists towards pharmacogenomics services.

**Methods:**

A cross-sectional study was conducted among community pharmacists in two cities in Northern Nigeria using a self-administered, validated and pre-tested questionnaire. The data were collected from December 2021 to February 2022 and were analysed using both descriptive and inferential analyses.

**Results:**

A total of 161 completed questionnaires were included in this study (response rate was 61.9%). Most of the respondents were males (59.0%). Only 25.5% had previous pharmacogenomics training but 90.1% indicated an interest in attending pharmacogenomics training in the future. Overall, respondents had moderate knowledge of pharmacogenomics with higher knowledge score found among those who had previous pharmacogenomics training (11.9 ± 1.7 vs 10.5 ± 2.4; *p* = 0.001), and those with postgraduate qualification (11.7 ± 1.9 vs 10.7 ± 2.3; *p* = 0.028). The mean attitude score was 6.8 ± 2.0 out of 10.0 indicating a good attitude towards pharmacogenomics services. Those with previous training (8.1 ± 1.7 vs 6.2 ± 1.9; *p* < 0.001) and those with postgraduate qualification (7.2 ± 2.3 vs 6.6 ± 1.9; *p* = 0.042) had better attitude towards pharmacogenomics services. The median perception score was 34.0 out of 45.0, indicating a positive perception towards pharmacogenomics. There was a better perception among those with previous pharmacogenomics training (40.0 [21–45] vs 34.0 [0–45]; *p* = 0.002) and those with postgraduate qualifications (39.0 [0–45] vs 34.0 [21–45]; *p* = 0.010). Barriers to the implementation of pharmacogenomics included lack of knowledge (89.4%), lack of guidelines (87.5%) and lack of reimbursement (81.4%).

**Conclusion:**

Community pharmacists have a moderate knowledge, a good attitude and a positive perception towards pharmacogenomics services. Those with previous pharmacogenomics training and those with postgraduate qualifications had better knowledge, attitude and perception towards pharmacogenomics services. Lack of knowledge, lack of guidelines and lack of reimbursement were the major barriers to the implementation of pharmacogenomics services in community pharmacies in Nigeria. Pharmacogenomics should be included in pharmacy training curricula to prepare pharmacists for the provision of pharmacogenomics services. Development of local guidelines and a robust reimbursement plan for pharmacogenomics services is recommended.

## Background

Pharmacogenomics is an important aspect in precision medicine. It involves the use of an individual’s genetic makeup to optimize pharmacotherapy by ensuring the efficacy of medications and minimizing adverse effects [1]. Pharmacogenomics testing services are used to optimize drug therapy and the results can be used to change drug therapy, adjust doses of medications, discontinue drug therapy and for monitoring treatment [2]. Initially, pharmacogenomics-tailored drug therapy was implemented in hospital settings [3] and hospital pharmacists played an important role in the implementation of the services in healthcare facilities [4]. Community pharmacists, the third largest healthcare professional group in the world after physicians and nurses [5], have the potentials to become the first healthcare professional that the public will contact for pharmacogenomics testing services [4]. The increasing public awareness regarding pharmacogenomics testing, continuous decrease in the cost of pharmacogenomics testing, and the implementation of direct-to-consumer testing have provided an opportunity for community pharmacists to incorporate pharmacogenomics services into patient care [4, 6]. Studies conducted in the United States (US), Canada and the Netherlands have demonstrated successful implementation of pharmacogenomics testing services in community pharmacy among post-myocardial infarction patients and patients taking antiplatelets such as clopidogrel [2–4, 6, 7].

The American Pharmacists Association supports the role of community pharmacists in pharmacogenomics services through medication therapy management. Community pharmacists have demonstrated their readiness to integrate pharmacogenomics services into patient care [2]. Community pharmacists offer a wide range of pharmacogenomics-related services including testing, counselling, interpreting results, and optimizing drug therapy (change of medication and dose modifications) based on pharmacogenomics results [2, 6, 7]. In developed countries such as the US, patients have demonstrated their interest to participate in pharmacogenomics services and they have shown their willingness to pay for the services [8]. In Canada, patients have identified community pharmacists as the ideal healthcare professionals to provide pharmacogenomics services [6]. There are some barriers that have hindered the application of pharmacogenomics in patient care and these include lack of knowledge and lack of self-confidence in the interpretation of genotyping results among community pharmacists [9–11]. Other barriers include lack of feasibility to provide pharmacogenomics services in community pharmacy, patients’ interest, resistance from prescribers and lack of a clear reimbursement policy [12]. However, these barriers are surmountable making the implementation of pharmacogenomics services in community pharmacy setting a possibility in the near future [12].

Community pharmacists are one of the most accessible healthcare professionals in Nigeria [13, 14]. The role of community pharmacists in Nigeria include dispensing medications [13], provision of pharmaceutical care services [15], vaccination and other health promotion services [15, 16]. There is paucity of data describing the application of pharmacogenomics in patient care owing to the lack of manpower, lack of equipped laboratories and lack of funding [17]. However, efforts are being made to implement pharmacogenomics-guided treatment in Nigeria. Currently, pharmacogenomics testing is being used to evaluate the prevalence of genetic polymorphism among people living with Human Immunodeficiency Virus (HIV) who are receiving highly active antiretroviral therapy [18, 19]. The findings from these ongoing studies are expected to guide the implementation of pharmacogenomics services into patient care. To understand the readiness of community pharmacists to offer pharmacogenomics services in Nigeria, their knowledge, attitude and perception towards pharmacogenomics services need to be explored. Previous studies have demonstrated limited knowledge, good attitude and positive perception towards pharmacogenomics services among pharmacists [10, 20–23]. However, studies involving among community pharmacists in Nigeria are lacking. Therefore, the objective of the current study is to evaluate the knowledge, attitudes, and perceptions towards pharmacogenomics services among community pharmacists in Nigeria.

## Methods

### Study design

This was a cross-sectional survey conducted among community pharmacists using a self-administered questionnaire.

### Study population and inclusion criteria

The study population included community pharmacists who practised in community pharmacies located in two cities in Northern Nigeria: Kaduna and Abuja. All practising community pharmacists were eligible for inclusion. Participation was voluntary and a cover letter enclosed in the questionnaire stated that the submission of a completed questionnaire is considered as consent to participate in the survey.

### Sample size estimation

The sample size was calculated using Raosoft Sample Size Calculator with the following assumptions: 5% margin of error, 95% confidence interval, 50% response distribution and the number of community pharmacists in the two states (*n* = 755) [24]. The calculated sample size for this study was 253 community pharmacists.

### Study instrument

The questionnaire used in this study was developed in English language after a review of previous literature [2, 12, 25–27]. It was validated by four lecturers who had expertise in pharmacogenomics and community pharmacy practice research, and the questionnaire was revised based on the comments and suggestions of the validators. The questionnaire consisted of 31 items and four sections including demographic section (seven items), knowledge (nine items), attitudes (five items) and perceptions (10 items) of community pharmacists towards pharmacogenomics services. Knowledge was assessed using ‘yes’, ‘no’ and ‘I don’t know’ while perceptions were assessed using a 5-point Likert scale (strongly agree, agree, neutral, disagree and strongly disagree). The validated questionnaire was pre-tested among 10 community pharmacists and the Cronbach’s alpha co-efficient was 0.629, 0.859 and 0.792 for knowledge, attitude and perception domain, respectively.

### Data collection

A snowball sampling technique was used to recruit community pharmacists to participate in the study. The investigators searched for community pharmacies in the two cities through signboards on buildings or in the streets. Community pharmacists recruited early on in the study were asked to help in identifying other community pharmacists in and around their area. The hardcopy version of the questionnaire was distributed to the community pharmacists by hand and the respondents were given the option to either complete the questionnaire on the spot or submit the completed questionnaire to the investigators at a mutually agreed date and time. The questionnaire took about 10–15 min to complete. Where there are more than one community pharmacists in a community pharmacy, all of them were invited to participate in the study. The data were collected from December 2021 to February 2022.

### Data analysis

The data were analysed using the Statistical Package for Social Sciences (SPSS) for Windows, version 22.0. Both descriptive and inferential analyses were conducted. Categorical variable was reported as frequency and percentage while continuous data were reported using either mean and standard deviation or median and range. Independent *T*-test and Mann–Whitney *U*-test (for 2 groups) and ANOVA and Kruskal–Wallis (three or more groups) were used to evaluate the difference in knowledge, attitude and perception scores based on the socio-demographic variables. Independent *T*-test and ANOVA were used for continuous and normally distributed data while Mann–Whitney *U*-test and Kruskal–Wallis tests were used for categorical data. *p* value less than 0.05 was considered as statistical significance. The items in the knowledge section were transformed into scores using zero and one point for wrong and correct answer, respectively. The items in the attitude section were transformed into scores (0 = no/not sure, 1 = yes, but after having had training on the subject, and 2 = yes, straight away). The total knowledge and attitude scores were computed as a sum of the points scored in each section. In the perception section, responses were transformed into scores using 5 points for strongly agree and 1 point for strongly disagree. Negative items were reverse-coded. The total perception score was computed and reported using median and interquartile range. The total knowledge, attitude and perception score was assessed using a scale of 0–17, 0–10 and 0–45, respectively, and the scores were transformed into percentages. The total knowledge was categorized using Bloom’s cut-off points into good (80–100%), moderate (60–79%) and low (below 60%) [28]. For attitude and perception, a modified Bloom’s cut-off scores less than 60% and ≥ 60 of the total score were considered as good/positive and poor/negative attitude and perception, respectively.

## Results

### Demographic characteristics of the respondents

A total of 161 community pharmacists completed the questionnaire and the response rate was 61.9%. Most of the respondents were males (59.0%) and aged between 20 and 29 years (42.2%) and had a Bachelor of Pharmacy (65.8%) as their highest qualification. Only 25.5% of the respondents had previous training in pharmacogenomics. However, an overwhelming majority (90.1%) indicated an interest in attending pharmacogenomics training in the future. Table 1 summarizes the characteristics of the community pharmacists who participated in the study.

**Table 1:** Demographic characteristics of the community pharmacists involved in the study

Variable	Frequency (*n* = 161)	Percentage (%)
Gender*		
Male	95	59.0
Female	60	37.3
Age group* (in year)		
20–29	68	42.2
30–39	53	32.9
40–49	24	14.9
50 and above	14	8.7
Years of working experience*		
Less than 5	74	46.0
5–10	56	34.8
11–15	5	3.1
More than 15	21	13.0
Highest academic qualification		
B. Pharm	106	65.8
PharmD	24	14.9
Masters	24	14.9
PhD	2	1.2
WAPCP	5	3.1
Position*		
Owner	23	14.3
Pharmacist	121	75.2
Manager	12	7.5
Previous pharmacogenomics training (yes)	41	25.5
Interest in attending pharmacogenomics training in future (yes)	145	90.1

*Denotes missing data, PhD: Doctor of Philosophy; WAPCP: West African Postgraduate College of Pharmacy

### Knowledge of pharmacogenomics among community pharmacists

Majority of the participants (> 90%) were aware that an individual’s genetic makeup affects the pharmacokinetics and pharmacodynamics of medications and that pharmacogenomics can be used to predict medication safety and efficacy. More than 50% of the respondents correctly identified paroxetine, simvastatin and warfarin as medications that require pharmacogenomics testing. However, only 21.1% and 29.8% were aware that pantoprazole and clopidogrel are affected by genetic variability, respectively. The mean total knowledge score was 10.9 ± 2.2 out of 17 and this indicates a moderate knowledge of pharmacogenomics among community pharmacists. Table 2 describes the knowledge of pharmacogenomics among community pharmacists.

**Table 2:** Number of respondents giving correct answers to questions assessing knowledge

Variable	Frequency (*n* = 161)	Percentage (%)
The pharmacokinetics and pharmacodynamics of a medication maybe affected by an individual’s genetic makeup	149	92.5
Pharmacogenomics testing can be used to predict medication safety and efficacy	150	93.2
Genetic determinants of drugs response may change over a person’s lifetime	14	8.7
Genetic variants account for most variability in drug disposition and effects	118	73.3
Pharmacogenomics test result can be used to change drug therapy	153	95.0
Pharmacogenomics test result can be used for dose adjustment	144	89.4
Pharmacogenomics test results can be used as an indication for discontinuation of a drug	146	90.7
Indication(s) for pharmacogenomics testing		
Therapeutic failure	74	46.0
To prevent adverse reaction	112	69.6
To guide initiation of therapy	137	85.1
Others		
Medications that require pharmacogenomics testing		
Paroxetine	98	60.9
Simvastatin	84	52.2
Cefuroxime*	77	47.8
Pantoprazole	34	21.1
Clopidogrel	48	29.8
Ibuprofen*	137	85.1
Warfarin	86	53.4

*Denotes medication that do not require pharmacogenomics testing; no is the correct answer

### Attitudes of community pharmacists towards pharmacogenomics

Around 40% of the respondents indicated an interest to provide pharmacogenomics services and recommend pharmacogenomics services to their patients straight away, while 42.2% indicated their willingness to offer pharmacogenomics services after they had undergone a training on this matter. Moreover, most community pharmacists (> 60%) indicated their willingness to receive and interpret pharmacogenomics testing results and advise patients on treatment choice based on the result after having had a training on pharmacogenomics. About 46% of the respondents indicated an interest in providing pharmacogenomics testing services straight away while another 49.1% were interested in providing the services after a training on the subject. Overall, the mean attitude score was 6.8 ± 2.0 out of 10.0 and this indicates a good attitude towards pharmacogenomics services. Table 3 describes the attitudes of community pharmacists towards pharmacogenomics services.

**Table 3:** Attitudes of community pharmacists towards pharmacogenomics services

Variable	Frequency (*n* = 161)	Percentage (%)
Would you recommend pharmacogenomics testing to your patients if those tests could predict that a specific drug could be efficacious in their case?		
Yes, straight away	69	42.9
Yes, but after having had training on the subject	68	42.2
No	15	9.3
Not sure	7	4.3
Are you willing to receive and interpret the pharmacogenomics testing results of your patients?		
Yes, straight away	56	34.8
Yes, but after having had training on the subject	101	62.7
Not sure	3	1.9
Would you advise your patient on a treatment choice based on patient’s pharmacogenomics testing result?		
Yes, straight away	52	32.3
Yes, but after having had training on the subject	106	65.8
Not sure	2	1.2
Would you make recommendations to a physician based on patient’s pharmacogenomics testing result?		
Yes, straight away	82	50.9
Yes, but after having had training on the subject	78	48.8
Are you interested in providing pharmacogenomics testing services?		
Yes, straight away	73	45.6
Yes, but after having had training on the subject	79	49.1
Not sure	8	5.0

### Perceptions of community pharmacists towards pharmacogenomics services

About 44% of the respondents strongly disagreed/disagreed that community pharmacists are well-placed within the healthcare system to provide pharmacogenomics services while 37.3% indicated strongly agreed/agreed. Most of the respondents (43.5%) indicated strongly agreed/agreed that pharmacogenomics testing services are feasible to be performed in community pharmacy setting. Most (89.6%) agreed that community pharmacists need training on pharmacogenomics. Similarly, 87.0% agreed that incorporation of pharmacogenomics into medication therapy management will optimize pharmacotherapy. About two-thirds (60.2%) agreed that pharmacogenomics testing will become a routine in clinical practice in the future. A lack of knowledge regarding pharmacogenomics (89.4%), lack of reimbursement for pharmacogenomics testing and services (81.4%) and lack of guidelines to interpret pharmacogenomics testing results (87.5) were the major barriers to the implementation of pharmacogenomics among community pharmacists. Overall, the median perception score was 34.0 out of 45 (range: 0–45) and this indicates a good perception towards pharmacogenomics services. Table 4 shows that perceptions of community pharmacists towards pharmacogenomics services.

**Table 4:** Perceptions of community pharmacists towards pharmacogenomics services

Variables	Frequency (%)				
	Strongly agree	Agree	Neutral	Disagree	Strongly disagree
Community pharmacists are well-placed within the healthcare system to provide pharmacogenomics services	29 (18.0)	31 (19.3)	29 (18.0)	31 (19.3)	40 (24.8)
Pharmacogenomics testing services is feasible in community pharmacy setting	34 (21.1)	36 (22.4)	38 (23.6)	40 (24.8)	12 (7.5)
Community pharmacists have the expertise to interpret and adjust medication doses based on patient’s pharmacogenomics results	36 (22.4)	50 (31.1)	42 (26.1)	12 (7.5)	20 (12.4)
Community pharmacists need training in pharmacogenomics	91 (56.5)	50 (31.1)	14 (8.7)	2 (1.2)	1 (0.6)
Physicians and community pharmacists should collaborate to offer pharmacogenomics testing	103 (64.0)	49 (30.4)	6 (3.7)	2 (1.2)	
Pharmacogenomics testing will prevent your patient from taking the inappropriate medicine or the wrong dose	97 (60.2)	49 (30.4)	11 (6.8)	2 (1.2)	1 (0.6)
Incorporation of pharmacogenetic screening into medication therapy management will optimize pharmacotherapy	65 (40.4)	75 (46.6)	15 (9.3)	3 (1.9)	
Pharmacogenomics testing will become a routine in clinical practice in the future	54 (33.5)	43 (26.7)	31 (19.3)	26 (16.1)	5 (3.1)
Pharmacogenomics-guided treatment is cost-effective	40 (24.8)	68 (42.2)	29 (18.0)	7 (4.3)	11 (6.8)
Barriers to implementation of pharmacogenomics services					
Lack of knowledge	86 (53.4)	58 (36.0)	8 (5.0)	2 (1.2)	2 (1.2)
Lack of reimbursement	71 (44.1)	60 (37.3)	16 (9.9)	5 (3.1)	2 (1.2)
Lack of time	63 (39.1)	53 (32.9)	25 (15.5)	10 (6.2)	3 (1.9)
Lack of guidelines	82 (50.9)	59 (36.6)	8 (5.0)	5 (3.1)	1 (0.6)
Ethical considerations regarding ownership of genetic data	77 (47.8)	44 (27.3)	28 (17.4)	2 (1.2)	3 (1.9)
Resistance from other healthcare professionals	74 (46.0)	52 (32.3)	24 (14.9)	4 (2.5)	1 (0.6)
Lack of acceptance by patients	78 (48.4)	40 (24.8)	22 (13.7)	8 (5.0)	7 (4.3)

### Difference in knowledge, attitude and perception scores among community pharmacists

There was no significant difference in total knowledge, attitude and perception scores based on years of working experience, and interest in attending pharmacogenomics training in the future. Community pharmacists with postgraduate qualifications had higher total attitude (7.2 ± 2.3 vs 6.6 ± 1.9; *p* = 0.042), total knowledge (11.7 ± 1.9 vs 10.7 ± 2.3; *p* = 0.028) and total perception (39 [0–45] vs 34 [21–45]; *p* = 0.010) scores than those with undergraduate qualification. Those with previous pharmacogenomics training had higher total attitude (8.1 ± 1.7 vs 6.2 ± 1.9; *p* < 0.001), total knowledge (11.9 ± 1.7 vs 10.5 ± 2.4; 0.001) and total perception (40 [21–45] vs 34 [0–45]; *p* = 0.002) scores than those who indicated no/I don’t know. Males were found to have significantly higher total perception score (36 [0–45]) than females (33 [21–45]). Community pharmacy managers had significantly higher total knowledge and total attitude scores than the others. The total attitude and total perception scores varied significantly based on the age of the respondents. Table 5 summarizes the difference in knowledge, attitude and perception score based on the demographics of the respondents.

**Table 5:** Difference in knowledge, attitude and perception score towards pharmacogenomics among community pharmacists

Variable	Total attitude score		Total perception score		Total knowledge score	
	Mean (SD)	*p* value	Median (range)	*p* value	Mean (SD)	*p* value
Gender						
Male	6.5 ± 1.9	0.621*	36.0 (0–45)	0.039^**^	10.8 ± 2.2	0.481*
Female	6.7 ± 2.1		33.0 (21–45)		10.9 ± 2.5	
Age group						
20–29	6.0 ± 1.5	** < 0.001**^**#**^	33.0 (21–45)	**0.013**^##^	10.4 ± 2.4	0.241^#^
30–39	6.8 ± 1.9		37.0 (26–44)		11.1 ± 2.4	
40–49	7.9 ± 2.0		32.5 (25–45)		11.2 ± 1.5	
50 and above	6.8 ± 2.9		38.5 (0–42)		11.8 ± 1.2	
Years of working experience						
≤ 10 years	6.6 ± 1.9	0.423*	34.0 (21–45)	0.086**	10.8 ± 2.4	0.187*
> 10 years	7.1 ± 2.5		36.0 (0–45)		11.4 ± 1.9	
Highest academic qualification						
Undergraduate degree	6.6 ± 1.9	0.042*	34.0 (21–45)	0.010**	10.7 ± 2.3	0.028*
Postgraduate degree	7.2 ± 2.3		39.0 (0–45)		11.7 ± 1.9	
Position						
Owner	7.2 ± 2.5	**0.004**^**#**^	37.0 (0–45)	0.103^##^	12.1 ± 1.7	**0.001**^#^
Pharmacist	6.5 ± 1.9		34 (21–45)		10.6 ± 2.4	
Manager	8.2 ± 1.5		38 (26–43)		12.1 ± 1.6	
Previous pharmacogenomics training						
Yes	8.1 ± 1.7	< 0.001*	40.0 (21–45)	0.002**	11.9 ± 1.7	0.001*
No/don’t know	6.2 ± 1.9		34.0 (0–45)		10.5 ± 2.4	
Interest in attending pharmacogenomics training in future						
Yes	6.7 ± 1.9	0.118*	35.0 (0–45)	0.123**	10.9 ± 2.2	0.346*
No/don’t know	5.3 ± 1.3		39.5 (33–41)		8.3 ± 4.9	

*T-test; **Mann–Whitney *U* test; ^#^one-way ANOVA test; ^##^Kruskal–Wallis test

Bold font denotes statistical significance

## Discussion

The current study evaluated the knowledge, attitude and perception of community pharmacists towards pharmacogenomics services. It was found that more than two-thirds of community pharmacists had no previous pharmacogenomics training, although an overwhelming majority indicated an interest in attending pharmacogenomics training in the future. Lack of knowledge and training in pharmacogenomics has been identified as one of the major barriers to the implementation of pharmacogenomics in clinical practice among healthcare professionals [9–11, 29]. The current study found that community pharmacists had moderate knowledge of pharmacogenomics and this does not corroborate the findings of previous studies conducted among pharmacists in Kuwait, Thailand and Saudi Arabia that reported a low knowledge of pharmacogenomics [25, 30, 31]. In addition, another study revealed that more than 50% of community pharmacists in the US lack knowledge of pharmacogenomics and need further training [20]. Furthermore, a systematic review also demonstrated a poor knowledge of pharmacogenomics among pharmacists and pharmacy students [10]. The inconsistency could be attributed to the increasing awareness about pharmacogenomics among healthcare professionals in recent years. In addition, about 35% of the population in the current study had a PharmD/postgraduate qualification and they may have been exposed to pharmacogenomics contents during their training resulting in a higher knowledge score compared to previous studies. Furthermore, undergraduate pharmacy students in Nigeria are exposed to some pharmacogenomics contents during their pharmacology lectures including the role of genetic polymorphism in explaining the variability in drug response among patients. However, pharmacogenomics and its application in the modification of drug therapy is not taught in undergraduate curriculum.

It was also found that more than two-thirds of the community pharmacists could not identify clopidogrel and pantoprazole as medications that need pharmacogenomics testing while one-third could not identify paroxetine, simvastatin and warfarin. This result is supported by a study conducted among Palestinian pharmacists which showed that more than two-thirds are not aware of medications that require pharmacogenomics testing [32]. Inadequate knowledge of pharmacogenomics can be attributed to lack of previous pharmacogenomics training among the respondents as less than one-third of the respondents in this study had previous training. This highlights the need for the inclusion of pharmacogenomics into pharmacy training curricula. In addition, pharmacogenomics training modules can also be designed for practising pharmacists and offered in the form of continuing professional development course or as a micro-credential course. A study conducted in the Netherlands demonstrated that an online pharmacogenomics training following by a workshop increased the knowledge and self-confidence of community pharmacists in delivering pharmacogenomics services [7]. Pharmacogenomics training should be standardized and a certificate of completion should be given to community pharmacists for display in their pharmacies to increase public trust and confidence in community pharmacist-led pharmacogenomics services [1]. The results showed that community pharmacists with previous pharmacogenomics training and those with postgraduate qualification had significantly higher knowledge score. A previous study involving hospital pharmacists in Saudi Arabia showed a higher knowledge of pharmacogenomics among those with postgraduate qualification [30]. This implies that community pharmacists with postgraduate qualification may be exposed to pharmacogenomics contents and evidence has shown that training increased the knowledge of pharmacogenomics among community pharmacists [7]. Therefore, pharmacogenomics should be included in undergraduate training curriculum to prepare future pharmacists for the provision of pharmacogenomics services.

The current study revealed that community pharmacists had a good attitude towards pharmacogenomics and this is consistent with the results of previous studies [10, 22, 23]. The respondents indicated their willingness to recommend pharmacogenomics testing, interpret pharmacogenomics test results, and make recommendations to both patients and physicians based on pharmacogenomics test results. A good attitude towards pharmacogenomics services reflects an awareness among community pharmacist regarding the application of pharmacogenomics in optimizing drug therapy and minimizing adverse effects. It was found that those with previous pharmacogenomics training and those with postgraduate qualification had a better attitude towards pharmacogenomics. These observations highlight the importance of pharmacogenomics training and postgraduate degree in improving the knowledge and attitude towards pharmacogenomics services among pharmacists. In addition, those aged between 40 and 49 years old had a higher total attitude score than the other age groups and this could be due to their higher years of work experience.

The current study found that community pharmacists had a positive perception towards pharmacogenomics services and this is similar to the finding of a previous study [21]. However, less than 50% of the respondents strongly agreed/agreed that community pharmacists are in the position to implement pharmacogenomics testing services and that pharmacogenomics testing is feasible in community pharmacy setting. This could be due to certain barriers that hinder community pharmacists from providing pharmacogenomics services. The results also showed that community pharmacists with previous pharmacogenomics training and those with postgraduate qualifications had better perception towards pharmacogenomics services. Again, this explains the impact of pharmacogenomics training and postgraduate degree on the perception of community pharmacists towards pharmacogenomics services. It is important to note that majority of the respondents indicated interest in attending pharmacogenomics training in the future. Pharmacogenomics training will be welcomed by community pharmacists if the training is accessible, available and inexpensive. Male respondents had better perception towards pharmacogenomics than females, most probably because there were more male respondents in the study. Also, respondents aged 50 years and above had better perception towards pharmacogenomics services than other age groups and this could be due to their higher years of working experience. In addition, older community pharmacists are more likely to have a postgraduate degree compared to younger community pharmacists. Additional studies are recommended to explain the impact of age and gender on pharmacists’ perception towards pharmacogenomics services.

There are several barriers that hindered community pharmacists from integrating pharmacogenomics into patient care. The major barriers identified in the current study include lack of knowledge, lack of guidelines, and lack of reimbursement for pharmacogenomics services. These barriers are similar to those reported in previous studies [10–12, 29]. Therefore, strategies to overcome these barriers are recommended to improve the participation of community pharmacists in pharmacogenomics services. These strategies should include pharmacogenomics training courses, workshops and seminars for practising pharmacists and the inclusion of pharmacogenomics into pharmacy training curricula to address the knowledge gaps. In addition, pharmacogenomics guidelines for testing and interpreting test results are recommended to facilitate the implementation of pharmacogenomics in community pharmacy settings. The Clinical Pharmacogenetics Implementation Consortium (CPIC) guidelines in the United States and the Dutch Pharmacogenetics Working Group recommendations are developed to facilitate the integration of pharmacogenomics services into patient care [33, 34]. These guidelines describe the medications and patients that need pharmacogenomics testing, the procedure for specimen collection and interpretation of the pharmacogenomics testing results [33, 34]. Most patients who seek community pharmacy services in Nigeria pay out of their pockets making reimbursement for the services difficult especially if the cost of the services is expensive. Therefore, a robust reimbursement policy is required including reimbursement for pharmacogenomics services through the National Health Insurance Scheme. In addition, public enlightenment about the clinical benefits of pharmacogenomics testing should be emphasized to improve public acceptance and increase patient’s willingness to pay for the services. In addition, direct-to-consumer pharmacogenomics services should be affordable to encourage acceptance among patients and enable them to pay for the services.

This study has a number of limitations, therefore, the results should be interpreted with caution. Firstly, a snowball sampling technique was used to identify community pharmacists in this study. There is a high possibility of missing some community pharmacists in both cities resulting in sampling bias. Secondly, the responses are self-reported and this is susceptible to social desirability bias. Thirdly, the study was conducted in two cities located in northern Nigeria and this may affect the generalizability of the results. Despite the limitations, the study provides an insight into the knowledge, attitude and perceptions of community pharmacists towards pharmacogenomics services in Nigeria and provides some clues on how to initiate the implementation of pharmacogenomics services in community pharmacy setting in Nigeria.

## Conclusion

Community pharmacists have a moderate knowledge, a good attitude and a positive perception towards pharmacogenomics services. Majority of community pharmacists indicated that they need training in pharmacogenomics and are willing to attend pharmacogenomics training in the future. Those with postgraduate qualification and those with previous pharmacogenomics training had better knowledge, attitude and perception towards pharmacogenomics services compared to those without previous training and those with undergraduate qualification. Lack of knowledge, lack of guidelines and lack of reimbursement for pharmacogenomics services were the major barriers to the implementation of pharmacogenomics services among community pharmacists.

## Data Availability

All data generated or analysed during this study are included in this published article.

## Abbreviations

SPSS: Statistical Package for Social Sciences
CPIC: Clinical Pharmacogenetics Implementation Consortium
WAPCP: West African Postgraduate College of Pharmacy
